# The updates and implications of cutaneous microbiota in acne

**DOI:** 10.1186/s13578-023-01072-w

**Published:** 2023-06-21

**Authors:** Cong Huang, Fan Zhuo, Baoquan Han, Wenting Li, Bin Jiang, Kaoyuan Zhang, Xingling Jian, Zhenzhen Chen, Hui Li, Haiyan Huang, Xia Dou, Bo Yu

**Affiliations:** 1 Department of Dermatology, Skin Research Institute of Peking University Shenzhen Hospital, Shenzhen Key Laboratory for Translational Medicine of Dermatology, Peking University Shenzhen Hospital, Shenzhen Peking University-the Hong Kong University of Science and Technology Medical Center, Shenzhen, 518036 China; 2 Department of Urology, Shenzhen University General Hospital, Shenzhen, 518055 China; 3 Biomedical Research Institute, Shenzhen Peking University-the Hong Kong University of Science and Technology Medical Center, Shenzhen, 518036 China

**Keywords:** Acne, Cutaneous microbiota, Pathogenesis, Probiotics

## Abstract

Acne is a chronic inflammatory skin disorder that profoundly impacts the quality of life of patients worldwide. While it is predominantly observed in adolescents, it can affect individuals across all age groups. Acne pathogenesis is believed to be a result of various endogenous and exogenous factors, but the precise mechanisms remain elusive. Recent studies suggest that dysbiosis of the skin microbiota significantly contributes to acne development. Specifically, *Cutibacterium acnes*, the dominant resident bacterial species implicated in acne, plays a critical role in disease progression. Various treatments, including topical benzoyl peroxide, systemic antibiotics, and photodynamic therapy, have demonstrated beneficial effects on the skin microbiota composition in acne patients. Of particular interest is the therapeutic potential of probiotics in acne, given its direct influence on the skin microbiota. This review summarizes the alterations in skin microbiota associated with acne, provides insight into its pathogenic role in acne, and emphasizes the potential of therapeutic interventions aimed at restoring microbial homeostasis for acne management.

## Introduction

Acne, a pervasive inflammatory skin disorder, is clinically characterised by seborrhea, noninflammatory and inflammatory lesions, along with potential scarring [1]. These acne lesions predominantly present on the face, neck, upper back, shoulders, and chest, correlating with the distribution and density of pilosebaceous units in acne patients [2, 3]. Recent studies provide growing evidence that dysbiosis—an imbalance of cutaneous microbiota—is implicated in the manifestation of inflammatory skin diseases, including acne [4–6]. Additionally, individuals with acne are more susceptible to be colonized by diverse microbiota, a phenomenon that has been associated with the clinical status of acne [4–6].

## Alterations in skin microbiota correlate with acne severity

The skin microbiomes of individuals with acne show significant alterations when compared to healthy controls [7]. Intriguingly, acne patients, particularly those with severe symptoms, demonstrate increased alpha-diversity and a higher proportion of four gram-negative bacteria, namely *Faecalibacterium*, *Klebsiella*, *Odoribacter*, and *Bacteroides*. These differences are not observed in patients with milder acne grades [7], implying a potential correlation between the composition of the skin microbiota and the severity of acne.

The overgrowth of *Cutibacterium acnes* (*C. acnes*, previously known as *Propionibacterium acnes*) has a long-standing association with acne pathogenesis [8, 9]. Recent metagenomic analyses have revealed that the strain structure of *C. acnes* in acne patients differs from that of healthy individuals, despite their similar relative abundances. Specifically, type IV and V strains are particularly prevalent in acne-affected skins [10], which suggests a potential correlation between specific *C. acnes* strains and acne pathology. In terms of overall *Cutibacterium* population, there is no significant difference between acne patients and healthy individuals. However, acne patients harboring antibiotic-resistant strains exhibit a greater quantity of *Cutibacterium* than patients without these strains [11]. Coincidentally, Barnard et al. have noted that acne patients exhibit a more diverse microbiome composition at both species and *C. acnes* strain levels, with an increase in virulence-associated factors [12]. This finding hints at a potential link between the virulent characteristics of skin microbiota and acne. Moreover, recent research has identified potential genetic determinants of *C. acnes* strains associated with acne [10]. This provides new evidence for the pathogenic mechanisms involving cutaneous microbiota. By comparing multiple *C. acnes* isolates from patients with moderate to severe acne and healthy controls, it is further demonstrated that antibiotic-resistant *C. acnes* strains are implicated in acne development [13]. This finding suggests that the susceptibility of host affects the clinical outcome of colonization. Together, these studies emphasize the intricate association between skin microbiota composition and acne severity.

## Endogenous risk factors contributing to skin microbiota dysbiosis in acne

Given the close association between acne severity and skin microbiota variations, it is crucial to consider the risk factors contributing to skin microbiota dysbiosis in acne. Generally, the unique microbiota colonization in acne-affected skin is influenced by multiple endogenous (primarily genetic factors, sex, skin site, etc.) and exogenous factors (including treatments like topical benzoyl peroxide, systemic antibiotics, and photodynamic therapy) [14–17].

### Impact of sex on the skin microbiota in acne patients

The study on the cutaneous microbiota in healthy individuals revealed differences between male and female. More recent studies further demonstrated variations in skin microbiota of the two sexes in terms of community structure and composition [18]. Overall, both the alpha- and beta-diversity analyses depicted a contrasting microbiota composition between males and females, with a greater bacterial diversity observed in women. Although the relative abundance of *Actinobacteria* was similar in both sexes, the secondary dominant phylum varied, with *Firmicutes* primarily present in male skins and *Proteobacteria* predominantly present in female skins [18]. Given that sex hormones contribute to skin homeostasis and acne pathogenesis, their role in impacting the skin microbiota in acne cannot be overlooked [19]. Interestingly, adult acne in women is not associated with a specific subtype of *C. acnes*, as opposed to teenage acne [20]. Nonetheless, this study did not compare the microbiota compositions between male and female acne patients of similar ages, a comparison that could provide insightful information for sex-specific acne treatment strategies.

### Microbial heterogeneity varies between skin sites in acne

The human skin is inhabited by distinct microbial communities that vary across different skin locations. Recent studies have revealed the heterogeneity in microbial distribution across skin sites in acne lesions and its association with disease severity [21–23]. For instance, alterations in skin microbiota are noted on the inflammatory skin of severe acne patients' backs, as well as on the faces of patients with mild to moderate acne [21]. These alterations, when compared to healthy individuals, suggest a correlation between the distinct microbial colonization across skin sites and acne severity. Particularly, changes in skin commensals, such as the *Propionibacteriaceae*, *Staphylococcaceae*, and *Enterococcaceae* families, have been observed [21]. These observations suggest their potential involvement in acne pathogenesis. *C. acnes*, a specific microbial species, is detected on the faces and backs of 71.4% of severe acne patients, contrasted to its presence in only 45.5% of healthy individuals [22]. Concurrently, acne patients exhibit a higher prevalence of phylotype IA1 (84.4%) in comparison to the healthy population. This phylotype is also predominantly found on the backs of acne patients [22]. However, a decrease in *C. acnes* phylotype diversity closely correlates with acne severity on the backs of acne patients [22]. These studies underscore the importance of considering site-specific variability when exploring the microbial heterogeneity in acne.

### Additional endogenous factors that influence cutaneous microbiota in acne patients

In addition to the factors previously noted, additional endogenous elements influence the cutaneous microbiota in acne patients. The phase of pubertal development, for instance, impacts the composition of the skin microbiome, as evidenced by the shift in microbial diversity observed between early and late puberty stages [24]. Certain *C. acnes* strains, specifically those within single locus sequence typing (SLST) A [IA_1_], D [IA_1_], and F [IA_2_] clusters, exhibit unique responses to pubertal stage and the presence of acne. Meanwhile, these strains exhibit a distinct acne-associated microbiome signature [24].

Furthermore, there is a documented correlation between the integrity of epidermal barrier and the skin microbiota in acne patients [25]. Individuals with acne typically display enhanced transepidermal water loss (TEWL) and reduced microbiome diversity in comparison to healthy subjects. The diversity of skin microbiota, as quantified by Shannon and Simpson diversity indices, shows negative correlation with both disease severity and TEWL, revealing the interplay between barrier function and cutaneous microbiota in acne patients [25].

Intriguingly, a greater prevalence of *Malassezia* is observed in noninflammatory lesions as opposed to inflammatory lesions in acne patients [26]. Concurrently, *Malassezia restricta* and *C. acnes* demonstrate similar proliferation patterns during the transition from noninflammatory to inflammatory lesions [26]. These observations suggest a potential role for shifts in fungal abundance during the transformation from non-inflammation to inflammation states.

## Therapeutic interventions change skin microbiota in acne patients

The skin microbiota in acne patients is not only influenced by endogenous factors as discussed above but also by external factors, particularly various types of treatment. A growing body of researches have demonstrated that differential shifts in the skin microbiota contingent on the treatment employed [5, 27]. Table 1 summarizes the alterations in skin microbiota caused by different acne treatments.

**Table 1:** Summary of changed microorganisms during different treatments in acne

Types of treatments	Disease status or severity	Study outcomes	Refs.
Benzoyl peroxide (BPO)	Teenagers with acne (aged 7–10 years) or preadolescent acne patients	The number and diversity of bacterial species decreased after BPO treatment, with the microbiome of treatment group more closely resembled those without acne. However, BPO treatment may damage the epidermal barrier in acne, which could be considered as side effect	[28–30]
Systemic antibiotic treatment	Moderate to severe	Oral minocycline administration improved the clinical outcomes, reduced *C. acnes* colonization, with variable changes in other specific bacterial populations. Meanwhile, the skin microbiota was enriched in probiotics following treatment	[31, 32]
		After doxycycline treatment, decreased clinical acne grades associated with reduced *C. acnes* abundance were observed. Additionally, doxycycline increased bacterial alpha-diversity in acne	[33]
Photodynamic therapy (PDT)	Severe acne	ALA-PDT treatment led to clinical improvements. Meanwhile, ALA-PDT treatment increased the diversity of skin microbiome, with decreased *C. acnes* abundance in severe acne	[42–44]
Retinoid	NA	Retinoid treatment improved the clinical acne grades, increased the alpha-diversity, and reduced the abundance of *Propionibacterium*, whereas increased the abundance of several other taxa, when compared with controls	[47]
Supramolecular salicylic acid (SSA)	Moderate-to-severe	The 30% SSA peels improved GAGS scores and skin barrier indicators, while decreased richness and evenness of cutaneous microbiome in acne patients	[49]
		The 2% SSA treatment increased the clinical outcomes, as well as the α- and β-diversity index in acne patients. Specifically, the relative abundance of *Staphylococcus*, *Ralstonia*, and *Streptococcus* was significantly decreased by 2% SSA treatment, with overall bacteria genera distribution tends toward the healthy status	[50]
Myrtacine^®^	Global Acne Severity Scale, GEA grades 2–3	The Myrtacine^®^-based cream improved acne lesions and reduced the level of erythromycin resistance *C. acnes* in acne patients, without changing the total *C. acnes* load	[52]

### Effects of topical benzoyl peroxide on microbiota composition in acne patients

Benzoyl peroxide (BPO) has been a long-standing, first-line topical treatment for acne [3]. Meanwhile, an increasing number of studies have demonstrated that BPO treatment modulates the skin microbiota in acne patients [27]. To investigate alterations in the microbiome following topical BPO treatment, a pilot study involved participants aged 7–10 years (with or without acne) was conducted [28]. The baseline data demonstrated a higher diversity of cutaneous bacteria in teenagers with acne compared to those without. Notably, post-BPO treatment, both the number and diversity of bacterial species diminished, with the microbiome of treatment group closely resembling that of participants without acne [28]. In contrast, despite a reduction in acne counts among preadolescent acne patients post-BPO treatment, Ahluwalia's study found the bacterial diversity of the skin microbiome to be comparable between pre- and post-treatment preadolescents [29], suggesting the limited impact of BPO on microbial alterations during acne treatment. Recent findings by Zhou et al. reveal that BPO treatment improved the Global Acne Grading System (GAGS) score and diminished porphyrin and red areas, whereas compromised the epidermal barrier function [30]. Further, a significant reduction in microbial diversity is observed post-treatment, compared to baseline data [30]. Therefore, while BPO treatment decreases GAGS score and reduces microbial diversity, it also damages the epidermal barrier in acne, which can be considered as a side effect.

### Impact of systemic antibiotics on cutaneous microbiota shift in acne

The application of antibiotics for acne treatment necessitates a comprehensive understanding of their effects on cutaneous microbial dysbiosis [5]. Chien et al. conducted a longitudinal cohort study to investigate the alterations in skin microbiota in response to antibiotic perturbation associated with acne treatment. Of all four acne patients prescribed oral minocycline, they observed an improvement in clinical outcomes, manifested by a reduction in *C. acnes* abundance post-treatment [31]. Concomitant with these findings, the study also reported distinct changes in other bacterial genera. Specifically, there was a transient increase in *Pseudomonas* species following antibiotic administration, a persistent increase in *Streptococcus* species, and a persistent decrease in *Lactobacillus* species, persisting up to eight weeks after minocycline withdrawal [31]. This study thereby demonstrates that systemic antibiotic treatment correlates with shifts in skin microbiota, characterized by variable changes in specific bacterial populations in acne. In a related study, Thompson et al. performed a case–control study to ascertain the impact of minocycline treatment on skin microbiota. Post-treatment, they observed an enrichment of probiotics *Bifidobacterium longum* and *Leuconostoc mesenteroides* within the skin microbiota, contrasted with a depletion of *Staphylococcus epidermidis* and *Prevotella nigrescens* [32]. At the phylum level, a decreased ratio of *Firmicutes* to *Bacteroidetes* in acne patients following treatment was detected [32]. This evidence suggests that minocycline treatment influences the composition of the acne skin microbiota, underscoring the potential benefits of developing more targeted antimicrobial strategies for acne.

To evaluate the alterations in skin microbiota structure and composition in acne patients following doxycycline treatment, a longitudinal cohort study was conducted on individuals with acne who were prescribed a six-week oral doxycycline [33]. Prior to the treatment, the dominant species was identified as *C. acnes*, which exhibited a positive correlation with the severity of acne [33]. Following doxycycline intervention, a decrease in clinical acne grades was observed, and this reduction was associated with a lower abundance of *C. acnes*. Furthermore, substantial variations were noted in other bacterial species such as *Cutibacterium granulosum*, which displayed increased abundance in the treated cohort [33]. Moreover, the administration of doxycycline resulted in an elevation of the bacterial alpha-diversity within the acne skin. In short, systemic antibiotics modify both the composition and diversity of acne microbiota, which in turn reflect the impact of antibiotic treatment.

### Antimicrobial susceptibility of *C. acnes* varies among acne patients

Systemic antibiotics, commonly prescribed for the treatment of acne, confer substantial benefits to patients. Nonetheless, the pervasive use of these antibiotics has sparked concerns regarding bacterial resistance, particularly in the case of *C. acnes* [23, 34]. Grech conducted a study investigating the susceptibility of *C. acnes* to amoxicillin, minocycline, erythromycin, and clindamycin using isolates obtained from acne patients. Notably, 37.8% of these isolates were resistant to both erythromycin and clindamycin, while a mere 4.4% exhibited resistance to all four antimicrobials [35]. Complementing these findings, Zhang et al. reported that the highest prevalence of resistance among clinical *C. acnes* strains was observed for erythromycin and clindamycin, with resistance rates of 49.2% and 28.6%, respectively [36]. Additionally, they found that the high resistance rates to clindamycin and erythromycin were significantly influenced by a history of macrolide treatment [37]. This finding implies that patients with prior exposure to macrolides should refrain from using clindamycin and erythromycin. Zhang et al. proceeded to investigate the draft genome sequences of multidrug-resistant *C. acnes* strains, thereby shedding light on potential genetic clue for antibiotic-resistance in specific strains of *C. acnes* [38]. Collectively, these studies provide valuable insights that can guide antimicrobial prescription for treating acne. Nevertheless, further in-depth studies with larger sample sizes are warranted to validate these findings.

### Impact of photodynamic therapy on cutaneous microbiota shift in severe acne

Photodynamic therapy (PDT) has been found to effectively improve clinical outcomes with favorable tolerability in the treatment of severe acne [39–41]. To investigate the impact of PDT on the diversity and composition of cutaneous microflora among severe acne patients, a study was conducted involving patients who were treated with 5-aminolevulinic acid-mediated PDT (ALA-PDT) once a week for three weeks. Healthy individuals were simultaneously recruited to serve as controls. The baseline data revealed marked differences in microbiota composition between healthy controls and acne patients, characterized by reduced alpha-diversity in the patient cohort [42]. Intriguingly, ALA-PDT treatment resulted in noticeable modifications to the patients’ microbiota composition, including 15 bacterial genera, such as *Enhydrobacter*, *Cetobacterium*, and *Streptococcus* [42]. In accordance with these findings, a recent prospective study demonstrated that ALA-PDT treatment served to enhance the diversity of the skin microbiome in acne patients [43]. Concurrently, ALA-PDT treatment suppressed the presence of *C. acnes* within the follicular microbiome, while increasing the abundance of resident follicular bacteria, predominantly *Bacillus* and *Lactococcus* [43]. This indicates that the therapeutic efficacy of ALA-PDT is partially attributed to its capacity to modulate the skin microbiome in acne cases. In support of this, Tao et al. reported a correlation between ALA-PDT administration and increased microbiota diversity in patients with severe facial acne [44]. Furthermore, their longitudinal cohort study provided evidence that ALA-PDT treatment contributed to clinical improvements, which were associated with a decrease in *C. acnes* colonization in severe acne patients [44]. Collectively, these findings suggest that the alterations observed in skin microbiota can serve as an indicator of the therapeutic efficacy of PDT in treating severe acne.

### Other treatments that affect microbiota shifts in acne skin

Systemic interventions, such as oral retinoids and tetracyclines, play significant roles in acne management owing to their anti-inflammatory properties [45, 46]. Notably, these treatments diminish the severity of clinical acne symptoms and the prevalence of *Cutibacterium*, while simultaneously increase the presence of various other taxa, including *Streptococcaceae*, *Pasteurellaceae*, and *Corynebacteriaceae*, relative to controls [47]. Prior to the treatments, no significant difference in alpha-diversity between control and acne patients is observed; however, a significant increase is noted post-treatment [47]. These findings suggest the potential of systemic treatments, other than antibiotics, to modulate the skin microbiota in individuals with acne.

Peels incorporating 30% supramolecular salicylic acid (SSA), a modified form of salicylic acid, have recently been demonstrated to provide a safe and effective treatment for moderate to severe acne [48]. To explore this treatment further, patients with acne were subjected to biweekly 30% SSA peels over a two-month period. Post-treatment, significant improvements were observed in GAGS scores and skin barrier indicators, alongside decreased richness and evenness of the cutaneous microbiome, and a reduced *Staphylococcus* proportion [49]. These findings indicate that 30% SSA peels can therapeutically benefit acne patients by modulating the skin microbiota. Furthermore, an investigation into the effect of 2% SSA on acne revealed significant improvements in clinical outcomes, as evidenced by decreased lesion counts and GAGS scores [50]. Specifically, the 2% SSA treatment resulted in increased alpha- and beta-diversity indices, reduced relative abundance of *Staphylococcus*, *Ralstonia*, and *Streptococcus*, and an overall shift in bacteria genera distribution toward a healthier state in acne patients [50]. Consequently, 2% SSA appears to normalize the microbial dysbiosis associated with acne-afflicted skin.

The plant-derived extract, Myrtus communis (Myrtacine^®^), is beneficial in acne treatment due to its anti-virulence and anti-inflammatory effects [51]. Notably, a cream formulated with Myrtacine^®^ significantly reduces the erythromycin-resistant (EryR) *C. acnes* population in acne patients [52]. Additionally, the Myrtacine^®^ cream improves acne lesions without altering the overall *C. acnes* load, suggesting its specific efficacy against EryR *C. acnes* [52].

## The regulatory roles of skin microbiota, particularly *Cutibacterium acnes*, in acne pathogenesis

**Fig. 1:**
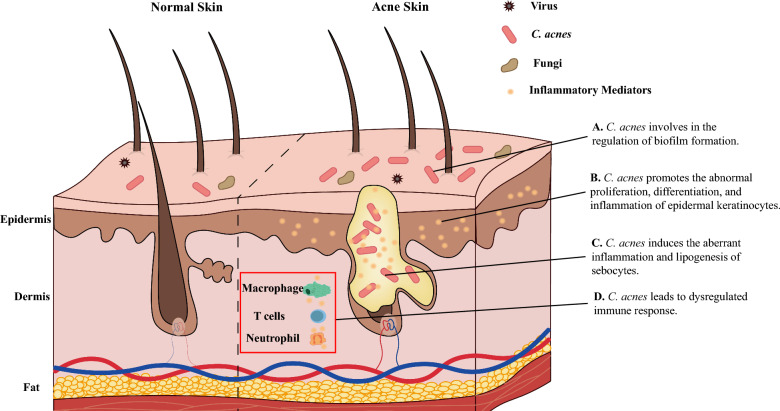
The regulatory roles of *C. acnes* in the pathogenesis of acne. *C. acnes* participates in the regulation of acne pathogenesis through multiple different ways. It involves in the regulation of biofilm formation (**A**); participates in the abnormal regulation of epidermal keratinocytes (**B**); regulates the aberrant inflammation and lipogenesis of sebocytes (**C**); and dysregulates the immune response (**D**)

Increasing evidence has implicated skin microbiota dysbiosis as a significant contributor to acne pathogenesis. Meanwhile, comprehensive researches have elucidated the impacts and molecular mechanisms of cutaneous microbiota, focusing predominantly on *C. acnes*, in the onset and progression of acne (Fig. 1).

### The influence of *C. acnes* on epidermal keratinocytes, biofilm formation, and immune regulation

*Cutibacterium acnes*, a gram-positive commensal bacterium, is a dominant species within the cutaneous microbiota and a crucial pathogenic factor in acne. This bacterium is involved in multiple pathways associated with acne pathogenesis. Its role in the regulation of keratinocytes' cell fate has been widely accepted, with several distinct mechanisms identified. Firstly, *C. acnes* has been found to stimulate epidermal keratinocyte proliferation via the IGF-1/IGF-1R axis, which correlates with increased expression of Ki67 and filaggrin [53]. Secondly, the bacterium influences keratinocyte differentiation by elevating levels of transglutaminase and keratin 17, while simultaneously reducing K1 and K10 levels in keratinocytes [54]. Thirdly, *C. acnes* has been reported to alter the barrier function of epidermal keratinocytes by modulating tight junction proteins and managing cell-to-cell contacts [55]. Fourthly, *C. acnes* has been implicated in the regulation of keratinocyte autophagy [56]. Further supporting this, propionic acid, a metabolite secreted by *C. acnes*, also contributes to autophagy in keratinocytes, underscoring the bacterium's profound influence on keratinocytes [56]. Lastly, *C. acnes* can trigger inflammatory responses in keratinocytes. Exposure to *C. acnes* results in a rapid production of superoxide anions in keratinocytes, associated with the release of pro-inflammatory molecules [57]. Moreover, keratinocytes coincubated with *C. acnes* instigate a pro-inflammatory response involving cytokines and chemokines, including IL-1β, granulocyte/macrophage colony-stimulating factor, and IL-8. This response implicates *C. acnes* in the recruitment of inflammatory cells to inflammation sites, thus facilitating acne lesion development [58]. Cumulatively, these studies demonstrate that *C. acnes* can shape acne pathogenesis through its substantial effects on keratinocyte proliferation, differentiation, barrier function, autophagy, and inflammation.

The role of bacterial biofilm formation in the pathogenesis of infections is crucial, and this has been particularly noted in the case of *C. acnes* both *in vitro* and *in vivo* [59–61]. A recent study examined the biofilm-forming characteristics of various *C. acnes* strains in acne patients and found that 23% of the acne specimens contained biofilm [62]. Biofilm was most frequently detected in comedones, present in 55.6% of specimens, whereas inflammatory papules and uninvolved skin had a lower frequency of detection at 22.2% each, among all the biofilm contained specimens [62]. This data suggests a potential correlation between biofilm formation by specific *C. acnes* strains and acne development. Interestingly, biofilm formation was also found to correlate with the phylotype of *C. acnes*, as different isolates showed variations in formed biofilm quantities [63]. Furthermore, different *C. acnes* phylotypes were observed to form structurally distinct biofilms and exhibit divergent adhesive properties [63]. Specifically, the phylotype IA1, which is more prevalent in acne-affected skin compared to healthy skin, displayed higher efficiency in early adhesion and biomass production than other phylotypes [64]. This implies a potential antibiotic tolerance, suggesting that novel antimicrobial agents targeting biofilm-forming *C. acnes* could serve as promising therapeutics for acne treatment. In acne lesions, the presence of biofilm-derived *C. acnes* activates miR-146a, TLR2 and its downstream pathways in keratinocytes [65]. Functionally, miR-146a inhibits the activation of TLR2 pathway mediated by *C. acnes*-derived biofilm [65]. This points toward the involvement of epigenetic regulation in the inflammation instigated by *C. acnes* biofilm and provides a novel clue for the *C. acnes* biofilm-mediated acne pathogenesis.

*Cutibacterium acnes* species derived from both acne lesions and healthy subjects did not show any distinct differences in cytokine production from peripheral blood mononuclear cells (PBMCs). However, the inflammatory cytokine production was markedly increased in PBMCs obtained from acne patients as opposed to those from healthy donors [66]. This finding suggests that the host response, rather than the bacterial species, serves as the crucial determinant of acne pathogenesis. Genomic studies have indeed unveiled the presence of both health-associated and acne-associated *C. acnes* strains in clinical contexts. This has considerably broadened our comprehension of the mechanisms involved in acne pathogenesis [10, 12, 67]. It was found that application of acne-associated *C. acnes* strains resulted in skin pathology in a murine model of acne, which was distinct from the effects observed with health-associated strains [68]. Moreover, acne-associated *C. acnes* strains elicited higher levels of inflammatory factors compared to their healthy counterparts [68]. Mechanistically, different *C. acnes* phylotypes induced distinctive immunological responses [69]. For example, acne-associated *C. acnes* phylotypes triggered higher levels of IFN-g and IL-17, while health-associated *C. acnes* phylotypes prompted a pronounced IL-10 response in PBMCs [69]. This provides evidence supporting a immunopathologic corroboration of health and disease association in *C. acnes* strains.

The host immune response toward *C. acnes* is also implicated in acne pathogenesis. Observations have been made of a substantial infiltration of CD4^+^ T cells in the perifollicular space of early acne lesions, further substantiating the role of T helper cells in the immune response prompted by *C. acnes* colonization [70]. In addition, IL-17-expressing cells were predominantly detected in lesional skins from acne patients. Furthermore, *C. acnes* robustly elicited a Th17 response in CD4^+^ T cells sourced from human PBMCs [70]. Importantly, supernatants from *C. acnes*-stimulated PBMCs effectively enhanced the differentiation of Th17 cells [70]. Consistently, PBMCs stimulated by acne-associated *C. acnes* strains manifested higher IL-17 levels as compared to those stimulated by *C. acnes* strains from healthy donors [71]. Interestingly, only health-specific Th17 clones secreted molecules with potent *C. acnes*-killing capabilities, with supernatants displaying strong antibacterial activity against *C. acnes* [71]. Hence, *C. acnes* strains from healthy or acne-ridden skin differentially modulate Th17 responses in acne. Supporting this, both Th17 and Th1 related cytokines and chemokines, along with their receptors, are notably upregulated in acne lesions [72]. Furthermore, *C. acnes* has been found to foster mixed Th17/Th1 responses by inducing the secretions of IL-17A and IFN-g from specific CD4^+^ T cells [72]. Intriguingly, *C. acnes*-specific Th17/Th1 cells are primarily found in the peripheral blood of acne patients [72], thus establishing these *C. acnes*-responding Th17/Th1 cells as a crucial CD4^+^ subpopulation implicated in acne pathogenesis.

In summary, *C. acnes* contributes to the pathogenesis of acne via several distinct mechanisms. These include the alteration of epidermal keratinocyte characteristics, the manipulation of biofilm formation, and the disruption of microbe-host immune interactions.

### *Cutibacterium acnes*-associated pathways that contribute to acne pathogenesis

*Cutibacterium acnes* is widely recognized as an etiological agent that propagates acne inflammation through various pathways. First, *C. acnes* instigates a robust immune response that involves the NLRP3-inflammasome during acne development. This response is evident as *C. acnes* induces the activation of monocyte-macrophage NLRP3-inflammasome and boosts the secretion of IL-1β in acne, thereby demonstrating its role in skin inflammation [73]. Corroborating this, human monocytes respond to *C. acnes* and secrete IL-1β partially through NLRP3-mediated pathway [74]. Notably, mature caspase-1 and NLRP3 are identifiable around the pilosebaceous follicles and macrophages within acne lesions, thus affirming the potential for *C. acnes*-mediated NLRP3 activation in acne development [74]. *C. acnes* can also stimulate the NLRP3 inflammasome in sebaceous glands, as evidenced by the detection of activated caspase-1 and IL-1β in human sebocytes exposed to *C. acnes* [75]. Moreover, knockdown of NLRP3 abolishes *C. acnes*-induced IL-1β production in sebocytes [75]. In addition, the silencing of NLRP3 hinders the production of IL-1β induced by *C. acnes* in sebocytes, and NLRP3-deficient mice exhibit a diminished inflammatory response to *C. acnes* [75]. This suggests that sebocytes are key immunocompetent cells and that *C. acnes*-induced NLRP3 activation in sebaceous glands plays a significant role in acne pathogenesis.

Second, *C. acnes* engages TLR2, a signaling molecule highly activated in acne lesions, and elicits inflammation in keratinocytes, sebocytes, and monocytes, thereby facilitating acne development [76, 77]. *C. acnes* exposure escalates TLR2 expression in human keratinocytes [78] and significantly induces hBD2 and IL-8 expression in cultured keratinocytes [79]. This induction can be attenuated by anti-TLR2 antibodies [79], signifying that inflammation stimulated by *C. acnes* is TLR2 dependent in keratinocytes. In human sebocytes, *C. acnes* extracts stimulate the expression of IL-8 and TLR2. However, knockdown of TLR2 or anti-TLR2 antibodies obstruct *C. acnes*-induced IL-8 production [80], highlighting the vital role of TLR2 signaling in *C. acnes*-mediated inflammation in sebocytes. In a mouse model of acne, mutation of the Christie-Atkins-Munch-Petersen factor (CAMP, a secretory factor of *C. acnes*) or vaccination with CAMP factor antibodies reduces *C. acnes* colonization and *C. acnes*-mediated inflammation [81]. Contrarily, purified CAMP factor 1 triggers the production of IL-8, which can be mitigated by TLR2 antibodies [82]. CAMP1-TLR2 binding intensity is strong in *C. acnes* strains that produce copious amounts of IL-8 [81], indicating a direct interaction between CAMP1 and TLR2. Clinically, acne lesions exhibit higher levels of CAMP factor and TLR2 than nonlesional skins [82], further substantiating that the CAMP factor of *C. acnes* is a key contributor to TLR2-related inflammation in acne.

Third, an increasing body of evidence underscores the significance of *C. acnes*-mediated activation of the aryl hydrocarbon receptor (AhR) pathway in acne pathogenesis [83, 84]. The AhR or selective AhR ligands manage lipid synthesis and differentiation in human sebocytes [85, 86]. Additionally, the AhR is able to modulate TLR2-mediated expression of TNF-α and IL-8 in human sebocytes [87], thereby highlighting its role in acne inflammation. Interestingly, AhR downstream CYP genes are upregulated by *C. acnes* in human sebocytes [88]. Simultaneously, *C. acnes* induces the nuclear translocation of the AhR protein and activates the AhR pathway. Moreover, *C. acnes* inhibits lipogenesis and promotes the differentiation of sebocytes, effects that are negated by AhR gene silencing [88], suggesting a non-acnegenic role of *C. acnes* in promoting acne remission via the AhR pathway.

### Factors that negatively regulate the *C. acnes*-induced inflammation in acne pathogenesis

Inflammation provoked by *C. acnes* is recognized as a pivotal factor in acne pathogenesis. Consequently, the identification of elements that deter this inflammation holds substantial potential for therapeutic intervention. Recent study reveal that Bmal1 and its downstream genes are suppressed in the skin of *C. acnes*-treated mice [89]. Furthermore, Bmal1 negatively regulates *C. acnes*-induced inflammation *in vitro* and *in vivo* [89], validating its repressive role in acne pathogenesis.

The TNFAIP3 interacting protein 1 (TNIP1), known to inhibit the NF-κB pathway, is rapidly induced in keratinocytes by *C. acnes* [90]. As such, TNIP1 acts to diminish NF-κB activation and the ensuing inflammatory response incited by *C. acnes* [90], establishing its role as a negative regulator of *C. acnes*-induced inflammation. Similarly, the tumor necrosis factor alpha-induced protein 3 (TNFAIP3), which inhibits TLR and NF-κB signaling, is induced by *C. acnes* in epidermal keratinocytes [91]. Concurrently, the TNFAIP3 expression is heightened in acne lesions relative to non-lesional skins. Notably, TNFAIP3 tempers the inflammation triggered by *C. acnes* in keratinocytes [91]. Recent evidence also implicates fibroblast growth factor 21 (FGF21) in exerting anti-inflammatory effects on the epidermal layer [92]. In keratinocytes, FGF21 acts to mitigate the activation of TLR2, NF-κB, and MAPK signaling prompted by *C. acnes* [92]. Moreover, FGF21 curbs the inflammation driven by *C. acnes* [92], suggesting its regulatory role in acne pathogenesis.

*Staphylococcus epidermidis* (*S. epidermidis*), an important constituent of the normal microflora and a beneficial skin commensal, has been found to cohabitate with *C. acnes* in acne lesions [93, 94]. Intriguingly, *S. epidermidis* represses *C. acnes*-induced inflammation [95]. Among the mechanisms involved, *S. epidermidis* facilitates glycerol fermentation, augmenting its inhibitory effects on *C. acnes* proliferation. Further, succinic acid, found in the fermented medium, efficaciously restricts *C. acnes* growth. In addition, the application of succinic acid significantly attenuates *C. acnes*-induced inflammation in mice [95]. Co-culture studies identified 30 out of 557 staphylococcal strains that displayed anti-*C. acnes* activities [94]. Remarkably, these strains selectively exclude acne-associated *C. acnes* phylotypes, favoring cohabitation with those healthy skin-associated phylotypes [94]. These strains also demonstrate selective antimicrobial activity against resilient *C. acnes* strains [96]. Furthermore, staphylococcal lipoteichoic acid mitigates inflammation induced by *C. acnes* [96], underlining its role in limiting inflammation and maintaining skin homeostasis.

### Roles of *C. acnes* derivatives in acne pathogenesis

*Cutibacterium acnes* derivatives significantly contribute to acne pathogenesis. For instance, extracellular vesicles originating from *C. acnes* (CEVs) stimulate acne-like phenotype in human keratinocytes [97]. Mechanistically, these CEVs modify the cellular properties of epidermal keratinocytes, thus facilitating acne pathogenesis through the induction of keratinocyte differentiation, inflammation, and proliferation [97].

Moreover, *C. acnes* produces various proteases that are integral to acne pathogenesis. These proteases induce inflammation *via* PAR-2 signaling. It is notable that both the protease activity and PAR-2 expression are heightened in acne lesions [98]. In addition, inhibition of serine protease or blockade of PAR-2 diminishes inflammation induced by *C. acnes* [98]. Further, PAR-2 aids in the differentiation and lipogenesis of sebocytes, processes mediated by *C. acnes* [99–101]. Thus, *C. acnes*-derived proteases are instrumental in acne pathogenesis.

Porphyrins produced by *C. acnes* also have a crucial role in the disease development of acne. There is a significant decrease in porphyrin levels in acne patients post-treatment, which correlates with clinical improvement [102]. Additionally, porphyrin production fluctuates among various *Cutibacterium* species, with *C. acnes* being the highest producer [103]. Importantly, porphyrin levels in different *C. acnes* strains can elucidate disease status: acne-associated strains produce higher porphyrin levels, particularly when supplemented with vitamin B12, in contrast to health-associated strains that yield fewer porphyrins and remain unresponsive to vitamin B12 [104]. Functionally, these porphyrins and the acne-associated *C. acnes* strains trigger inflammation in keratinocytes [105, 106]. Furthermore, porphyrins or the acneic strains stimulate K^+^ leakage and activate NRLP3 inflammasome in keratinocytes. Notably, both porphyrin production and IL-1β release are higher in acne-associated strains [106]. A repressor gene of porphyrin biosynthesis, deoR, has been identified in health-associated *C. acnes* strains [103, 104], suggesting a novel mechanism in the pathogenesis of acne.

Additionally, propionic acid, a metabolite secreted by *C. acnes*, is known to exert deleterious effects when its local concentration surges due to excessive growth of *C. acnes* [107], providing insights into the dual role of *C. acnes* in maintaining healthy skin and facilitating pathogenic conditions.

In summary, extracellular vesicles, proteases, and metabolites derived from *C. acnes* collectively facilitate acne pathogenesis *via* numerous distinct mechanisms.

## Therapeutic strategies targeting skin microbiota (especially *C. acnes*) in acne treatment

As discussed previously, *C. acnes* is implicated in acne pathogenesis by triggering hyperproliferation and inflammation in keratinocytes, mediating abnormal biofilm formation, and dysregulating sebocyte lipogenesis. Thus, interventions targeting pathogenic *C. acnes* introduce a novel frontier in anti-acne therapy.

### Implications of natural products/molecules targeting *C. acnes* in acne treatment

Increasing evidence suggests that natural products and molecules possess substantial potential for acne treatment by targeting *C. acnes*-induced pathology (Table 2). For instance, Toona sinensis, traditionally used to manage enteritis and pruritus, exhibits antibacterial and anti-inflammatory effects on *C. acnes*-infected keratinocytes [108], indicating its potential use in acne treatment. Nicotinamide, a proven therapeutic agent for acne inflammation, attenuates inflammatory IL-8 production in *C. acnes*-stimulated keratinocytes [109]. Recently, piceatannol (3, 5, 3′, 4′-tetrahydroxy-trans-stilbene, PCT), a natural dietary component, has been noted for its role in mitigating acne by inhibiting *C. acnes*-mediated cell proliferation and inflammation [110]. Likewise, Orobol (3′,4′,5,7-tetrahydroxyisoflavone), a metabolite of genistein, suppresses NF-κB and MAPK signaling, and reduces expression of the proliferation marker Ki67 in *C. acnes*-induced keratinocytes [111]. Thus, both PCT and Orobol alleviate *C. acnes*-prompted inflammation and hyperkeratinization, presenting potential utility in acne treatment.

**Table 2:** Summary of natural products/molecules targeting *C. acnes* in acne treatment

Names of natural products/molecules applied	Experimental model/Clinical study	Functions	Refs.
Toona sinensis extract	*C. acnes*-treated HaCaT cells	Its extract shows antibacterial and anti-inflammatory effects on *C. acnes*-induced keratinocytes	[108]
Nicotinamide	HaCaT cells and primary keratinocytes stimulated by *C. acnes*	Nicotinamide decreases inflammatory IL-8 production in *C. acnes*-stimulated keratinocytes	[109]
Piceatannol (PCT) and Orobol	*C. acnes*-induced HaCaT keratinocytes	PCT and orobol alleviate the inflammation and hyperkeratinization mediated by *C. acnes* in keratinocytes	[110, 111]
Licochalcone A	*C. acnes*-treated primary mouse macrophages and human SZ95 sebocytes, and *C. acnes*-induced skin inflammation in mice	It blocks *C. acnes*-induced inflammation in macrophages and sebocytes. Moreover, its topically application attenuates *C. acnes*-induced skin inflammation in mice	[112]
Schisandrin A, B, and C	*C. acnes*-infected THP-1 cells	Schisandrin A, B, and C inhibit *C. acnes*-induced pyroptosis and inflammation *via* NLRP3 pathway	[113]
Baicalin and Polyphyllin I	*C. acnes*-induced THP-1 cells and HaCaT cells, and *C. acnes*-injected rats used as the acne model	Baicalin and Polyphyllin I alleviate *C. acnes*-induced inflammation through modulating the NLRP3 pathway	[114–116]
SIG1273 and SIG1459	Human keratinocytes exposed to *C. acnes*, a randomized and double-blind controlled trial, and a vehicle controlled head-to-head comparison between SIG1459 and 3% BPO	Both SIG1273 and SIG1459 combat against *C. acnes*. Meanwhile, both of them improve the clinical outcome of acne, with well tolerance. Moreover, 1% SIG1459 outperforms 3% BPO in a head-to-head comparison against BPO	[117, 118]
Myricetin	*C. acnes*-stimulated human SZ95 sebocytes	Myricetin inhibits the *C. acnes*-stimulated inflammation in sebocytes via suppressing the TLR2 and rapamycin pathways	[119]
Quercetin	*C. acnes*-stimulated HaCaT, THP-1 and RAW 264.7 cells, and *C. acnes*-induced skin inflammation in mice	Quercetin suppresses the *C. acnes*-mediated inflammation via inhibiting the TLR-2 and MAPK pathways in vitro. *In vivo*, quercetin reduces mouse cutaneous erythema and swelling induced by *C. acnes*	[120]
The extract of Helichrysum odoratissimum (L.) Sweet	*C. acnes*-induced HaCaT cells	It prevents the biofilm formation of *C. acnes*, controls *C. acnes* proliferation, and exhibits inhibitory activity on factors associated with bacterial virulence	[121]
*Arctostaphylos uva-ursi* leaf extract	HaCaT cells and HaCaT cells cotreatment with heat-killed *C. acnes*	It decreases the *C. acnes*-induced inflammation. Moreover, it disrupts the biofilm formation of *C. acnes* without affecting keratinocyte growth	[122]
3,3'-diindolylmethane (DIM)	Planktonic cells/NA	DIM inhibits biofilm formation by *C. acnes* without affecting the viability of cell growth. Also, DIM inhibits the biofilm formation of multiple other species. Moreover, DIM inhibits the expression of biofilm-related genes in *C. acnes*	[123]
G2 dendrigraft of lysine dendrimer (G2)	Human skin explants	G2 modifies the biofilm formation of *C. acnes*. Additionally, G2 decreases the inflammation and improves skin desquamation after *C. acnes* colonization. Moreover, G2 increases the diversity of *C. acnes*, with a modification of the balance between *C. acnes* phylotypes	[124]
*Kaempferia parviflora*	*C. acnes*-stimulated HaCaT cells and IGF-1 induced sebocytes	*Kaempferia parviflora* modulates the inflammatory signals in *C. acnes*-stimulated HaCaT cells and inhibits the lipogenesis of sebocytes	[125]
Mangifera indica leave	Sebocytes and sebaceous glands from skin explants	It reduces the *C. acnes* lipase activity from a severe acne phylotype. Additionally, it protects the microbiota equilibrium	[126]
Bee venom (BV) and melittin	Models of IGF-1 or *C. acnes*-induced lipogenic skin disease	In the *C. acnes*-induced mouse model, BV and melittin decrease the transcriptions of genes involved in lipid biosynthesis and inflammation mediated by *C. acnes*	[127, 128]

The *C. acnes*-induced NLRP3 inflammasome activation is critical for triggering inflammation and aggravating acne progression. Therefore, natural products/molecules targeting this pathway represent innovative approaches to acne treatment. For instance, Yang et al. reported that licochalcone A, a chalconoid derived from Glycyrrhiza inflate, effectively inhibits the *C. acnes*-activated NLRP3 inflammasome [112]. Additionally, licochalcone A suppresses *C. acnes*-induced production of caspase-1 and IL-1β in macrophages and sebocytes, and topical application of this compound attenuates *C. acnes*-induced skin inflammation in mouse models [112], signifying clinical applicability for acne treatment. Schisandrin A, B, and C, representative lignans of Schisandra chinensis Baill., counteract *C. acnes*-induced pyroptosis and inflammation, notably by attenuating IL-1β secretion and pyroptosis mediated by NLRP3 activation [113]. This evidence underscores their potential as promising therapeutic agents for acne. Furthermore, baicalin, a lipophilic flavonoid glycoside from *Radix Scutellariae*, also reduces skin inflammation through inhibiting NLRP3 activation [114]. Finally, Polyphyllin I, a steroidal saponin derived from Paris polyphylla rhizomes, has been demonstrated to alleviate *C. acnes*-induced inflammation, in part by downregulating NLRP3 pathway [115, 116], thus implying its therapeutic potential for managing acne inflammation.

*C. acnes* stimulates an innate immune response through activation of TLR2 signaling, a pivotal process in comedogenesis, and a significant factor in acne pathogenesis [117]. The isoprenylcysteine molecule, SIG1273, has been shown to inhibit TLR2 pathway and kill *C. acnes*, offering dual benefits for acne-affected skin [118]. Results from a double-blind controlled trial further demonstrate that SIG1273 gel improves the clinical outcomes for acne patients and is well-tolerated, suggesting its potential application in the treatment of acne [118]. More recently, SIG1459, another anti-acne isoprenylcysteine molecule, demonstrated the ability to counteract *C. acnes* and inhibit TLR2 signaling [117]. Additionally, 1% SIG1459 exceeded the performance of 3% BPO in a comparative clinical study, revealing its potential as a promising and safe acne treatment [117]. Myricetin, an extract commonly found in traditional Asian medicine, mitigates *C. acnes*-stimulated inflammation in sebocytes by suppressing TLR2 and rapamycin pathways activated by *C. acnes*, suggesting its potential in acne treatment [119]. Quercetin, a widely recognized plant polyphenolic antioxidant, attenuates *C. acnes*-induced inflammation by inhibiting TLR2 and MAPK pathways in HaCaT and THP-1 cells [120]. Furthermore, quercetin significantly reduces cutaneous erythema and swelling triggered by *C. acnes* in mouse models [120], indicating its therapeutic value in treating acne.

*C. acnes* biofilm formation is implicated in acne pathogenesis, and blocking this process represents a novel therapeutic approach [59–61]. The methanolic extract of Helichrysum odoratissimum (L.) Sweet targets bacterial growth while concurrently inhibiting *C. acnes* biofilm formation, highlighting its potential as a comedolytic agent for acne treatment [121]. *Arctostaphylos uva-ursi* leaf extract, a natural product, has demonstrated a bacteriostatic action against *C. acnes*-induced inflammation [122]. Most importantly, this extract disrupts *C. acnes* biofilm formation without affecting keratinocyte growth [122]. Indoles are ubiquitous molecules in both prokaryotes and eukaryotes. Of the 20 indoles that have been tested, indole-3-carbinol and 3,3′-diindolylmethane (DIM) have been demonstrated to significantly inhibit *C. acnes* biofilm formation without altering cellular viability [123]. Also, DIM successfully inhibits the biofilm formation by multispecies, including *C. acnes*, *S. aureus*, and *C. albicans*. Transcriptional analyses further reveal that DIM suppresses the expression of biofilm-related genes in *C. acnes*, confirming its property in blocking the biofilm formation of *C. acnes* and suggesting its utility in acne treatment [123]. Recently, Attia-Vigneau et al. identified a G2 dendrigraft of lysine dendrimer (G2) capable of modifying membrane fluidity and biofilm formation in *C. acnes* [124]. Notably, G2 ameliorated inflammation and enhanced skin desquamation following *C. acnes* colonization [124]. Moreover, G2 treatment diversified *C. acnes* phylotypes, indicating that the incorporation of such compounds in cosmetic products could be a novel strategy for acne prevention.

Sebocyte dysfunction, mediated by *C. acnes*, contributes to acne pathogenesis. Notably, the main component of *Kaempferia parviflora*, a traditional health-promoting medicine, has been shown to inhibit sebocyte lipogenesis [125]. Additionally, *Mangifera indica* leave, a previously reported anti-acne agent, also decrease *C. acnes* lipase activity, hinting at their potential roles in acne treatment [126]. Bee venom (BV) and melittin, known for their antibacterial, antiviral, and anti-inflammatory activities in various cell types, have been found to mitigate the upregulation of genes involved in lipid biosynthesis and inflammation triggered by *C. acnes*. This indicates the potential of BV and melittin as natural anti-acne agents targeting *C. acnes*-induced abnormal lipogenesis [127].

### Implications of next-generation antibiotics in acne treatment

The development of resistant *C. acnes* strains poses a significant challenge to the efficacy of current antibiotics in acne treatment, prompting urgent consideration in dermatology. Interestingly, isotretinoin, a non-antimicrobial retinoid, is shown to be effective in reducing the anaerobic bacteria *C. acnes* without antibiotic activity [128]. Orally administered isotretinoin displays satisfactory efficacy in moderate to severe acne, corresponding with the reduction in antibiotic-resistant *C. acnes* on the skin, hence suggesting its potential as an alternative to current antibiotic use [128].

VB-1953 is a next-generation antibiotic with bactericidal activity against resistant *C. acnes* strains. A recent study by Batra et al. showed that topical application of 2% VB-1953 gel resulted in substantial decrease in both inflammatory and noninflammatory lesion counts compared to the baseline [129]. In addition, VB-1953 treatment dramatically reduced resistant bacterial populations, specifically clindamycin-resistant *C. acnes* [129]. The study also reported minimal adverse events [129], affirming VB-1953 as a safe and effective therapy for acne involving resistant *C. acnes* strains.

Immunization with heat-inactivated *C. acnes* vaccines offers a novel therapeutic approach to acne. These vaccines have been shown to protect mice against *C. acnes* challenges and to suppress *C. acnes*-induced skin inflammation [130]. Furthermore, the vaccines effectively neutralize *C. acnes* cytotoxicity and attenuate inflammation in human sebocytes [130]. Thus, vaccination against cytotoxic skin bacteria represents a novel therapeutic for acne.

CBT-SL5, an antimicrobial peptide from Enterococcus faecalis SL5, exhibits antimicrobial activity against *C. acnes* [131]. Importantly, CBT-SL5 treatment diminishes *C. acnes*-induced inflammation by inhibiting NF-κB activation [132]. A randomized, placebo-controlled, split-face comparative study demonstrated that acne severity improved significantly on the side of the face treated with CBT-SL5 compared to the control side (treated with vehicle lotion) after 4 weeks [133]. Additionally, the phylogenetic diversity of the skin microbiota was reduced on the treated side [133], pointing CBT-SL5 as a promising antimicrobial option for acne treatment.

In short, next-generation antibiotics have the potential to provide an alternative choice, enhance the effectiveness of current antibiotics, and address the challenge of antibiotic resistance in acne treatment.

### Implications of probiotics and postbiotics in acne treatment

Probiotics and postbiotics, which constitute a segment of viable microbial dietary supplements, have demonstrated beneficial effects in combating pathogens and preserving the balance of skin microbiota. They also serve as adjuvant therapies complementing traditional acne treatments [134–136].

In a comprehensive study leveraging functional screening, genetic analysis, and proteomics, O'Neill et al. identified a particular strain of *Staphylococcus capitis* (S. capitis E12) that selectively inhibited *C. acnes* growth [137]. Notably, the potency of S. capitis E12 surpassed that of commonly prescribed antibiotics without exhibiting any toxicity to human keratinocytes or impacting other commensal skin bacteria [137]. This suggests the potential for utilizing skin microbiome in a biotherapeutic approach toward acne treatment.

The non-acne-causing strains can regulate the skin microbiome, leading to a decline in acne severity, thereby suggesting their therapeutic potential in acne treatment [138]. In a pilot study, Karoglan et al. demonstrated that the application of these non-acne-causing strains led to an improvement in comedone counts [138]. Following treatment, the skin microbiome composition in acne patients shifted toward the study formulations, with no adverse effects or flare-ups, confirming the safety and efficacy of these non-acne-causing strains [138]. Specifically, select strains of *actobacilli* have been shown to decrease inflammatory lesions in patients with mild to moderate acne [139]. The application of these selected *Lactobacilli* strains led to a temporary modulation of the skin microbiome, including a decrease in the abundance of *C. acnes* and an increase in *Lactobacilli* [139]. Notably, the reduction in inflammatory lesions was sustained for over four weeks post-lactobacilli application. These findings suggest the use of a specific *Lactobacilli* strain as a feasible therapeutic strategy for acne.

As outlined in “Factors that negatively regulate the C. acnes-induced inflammation in acne pathogenesis” section, *S. epidermidis* has been proven to inhibit *C. acnes* growth and attenuate *C. acnes*-induced inflammation [95], indicating its potential for the development of probiotics for acne. Recent findings have demonstrated that polyethylene glycol (PEG)-8 Laurate, a carbon-rich compound, selectively enhances the fermentation of *S. epidermidis*, thereby amplifying its probiotic effect against acne [140]. The application of PEG-8 notably reduced *C. acnes* growth and associated inflammation, and potentiated the anti-*C. acnes* activity of clindamycin [140]. Thus, the fermentation of *S. epidermidis* can serve as a probiotic strategy against *C. acnes*, thereby minimizing the reliance on antibiotics. Furthermore, when *S. epidermidis* was incubated with 2% PEG-8 Laurate, electricity was generated, resulting in significant growth retardation and cell lysis of *C. acnes* [141]. Additionally, the electricity generated using the *S. epidermidis* and PEG-8 Laurate mixture substantially inhibited the overgrowth of *C. acnes* in mouse models [141]. Nonetheless, the direct application of live *S. epidermidis* as a probiotic carries the risk of bloodstream infections. To mitigate this risk, Yang et al. developed polysulfone microtube array membranes (PSF MTAM) to encapsulate the probiotic *S. epidermidis* [142]. The encapsulated *S. epidermidis* enhanced the glycerol fermentation of *S. epidermidis* without any leakage [142], thus positioning it as a secure probiotic patch for acne treatment.

A previous study demonstrated that the *Weissella viridescens* UCO-SMC3 strain hindered the growth of *C. acnes* [143]. Moreover, this UCO-SMC3 strain manifests both antimicrobial and immunomodulatory capabilities, decreasing the adhesion of *C. acnes* and modulating the immune response to this bacterial infection [144]. A pilot study further substantiated these findings, indicating that a facial cream incorporating the UCO-SMC3 strain significantly mitigate acne lesions, thereby corroborating its advantageous use as a probiotic in acne treatment [144].

To compare the effectiveness of a probiotic derived from *Lactobacillus paracasei* versus 2.5% BPO in treating mild to moderate acne, Sathikulpakdee et al. conducted a randomized controlled trial. Following a four weeks’ treatment, a significant decrease in both inflammatory acne lesions and erythema index was noted in relation to baseline metrics in both the probiotic and BPO groups, with no substantial difference discerned between the two cohorts [145]. This supports the proposition that a probiotic-derived lotion could effectively treat mild to moderate acne, yielding outcomes comparable to those achieved with 2.5% BPO.

The use of skincare cosmetics containing anti-acne postbiotics has also been identified as a potent modality for acne mitigation [146]. A notable improvement in acne lesions was observed following two weeks of postbiotic treatment when compared with baseline measurements. In addition, postbiotics were found to bolster skin barrier functions, as manifested by a reduction in TEWL and sebum production. These results suggest that postbiotics could offer a promising therapeutic avenue for acne reduction [146].

## Prospects and perspectives

The dysbiosis of skin microbiota is increasingly being recognized as a crucial mechanism in the progression of acne. More specifically, a substantial correlation exists between the increased colonization of *C. acnes* and the severity of acne disease. Concurrently, treatments that target the skin microbiota, particularly *C. acnes*, are emerging as novel strategies for acne treatment. While numerous natural products, molecular compounds, and probiotics have demonstrated considerable potential in treating acne, the precise mechanisms underlying their efficacy remain to be elucidated, thereby presenting several obstacles to their improved clinical applications:

1. The majority of existing studies exploring the link between skin microbiota and acne have relied on cell-based or mouse models, with very few based on early-phase clinical trials. Therefore, significant further research is required to enable effective clinical implications.
2. The composition of skin microbiota is susceptible to both endogenous and external influences. Yet, existing research primarily investigates the impact of a single or a couple of factors on the dysbiosis of skin microbiota in acne pathogenesis. Consequently, it is imperative to establish a systematic model to examine skin microbiota alterations under various conditions. More importantly, we must comprehensively view the skin microbiome as a holistic entity involved in the pathogenesis and/or treatment of acne.
3. A multitude of natural products currently display potential for targeting *C. acnes* and mitigating acne. However, the complexity of some natural products' components can lead to severe side effects. Thus, it is important to carefully isolate the beneficial components and reevaluate their effects on acne treatment.
4. *C. acnes* is a widely known pathogenic factor in acne development. However, researchers have perhaps overly concentrated on its regulatory roles in acne pathogenesis over the past decades. Therefore, it is vital to expand our investigations to include other species associated with acne pathogenesis apart from *C. acnes*.

## Data Availability

Not applicable.

## Abbreviations

*C*. *acnes*: *Cutibacterium* *acnes*
TEWL: Transepidermal water loss
BPO: Benzoyl peroxide
GAGS: Global Acne Grading System
PDT: Photodynamic therapy
ALA-PDT: 5-aminolevulinic acid mediated PDT
SSA: Supramolecular salicylic acid
EryR: Erythromycin resistance
PBMCs: Peripheral blood mononuclear cells
CAMP: Christie-Atkins-Munch-Petersen
AhR: Aryl hydrocarbon receptor
TNFAIP3: Tumour necrosis factor alpha-induced protein 3
FGF21: Fibroblast growth factor 21
*S.**epidermidis*: *Staphylococcus* *epidermidis*
PCT: Piceatannol
G2: G2 dendrigraft of lysine dendrimer
BV: Bee venom
PEG: Polyethylene glycol

## References

[1] Ramasamy S, Barnard E, Dawson TL, Li H (2019). The role of the skin microbiota in acne pathophysiology. Br J Dermatol.

[2] Kraft J, Freiman A (2011). Management of acne. CMAJ.

[3] Eichenfield DZ, Sprague J, Eichenfield LF (2021). Management of acne vulgaris: a review. JAMA.

[4] O'Neill AM, Gallo RL (2018). Host-microbiome interactions and recent progress into understanding the biology of acne vulgaris. Microbiome.

[5] Xu H, Li H (2019). Acne, the skin microbiome, and antibiotic treatment. Am J Clin Dermatol.

[6] Dréno B, Dagnelie MA, Khammari A, Corvec S (2020). The skin microbiome: a new actor in inflammatory acne. Am J Clin Dermatol.

[7] Li CX, You ZX, Lin YX, Liu HY, Su J (2019). Skin microbiome differences relate to the grade of acne vulgaris. J Dermatol.

[8] Dessinioti C, Katsambas AD (2010). The role of Propionibacterium acnes in acne pathogenesis: facts and controversies. Clin Dermatol.

[9] Beylot C, Auffret N, Poli F, Claudel JP, Leccia MT, Del Giudice P (2014). Propionibacterium acnes: an update on its role in the pathogenesis of acne. J Eur Acad Dermatol Venereol.

[10] Fitz-Gibbon S, Tomida S, Chiu BH, Nguyen L, Du C, Liu M (2013). *Propionibacterium*
*acnes* strain populations in the human skin microbiome associated with acne. J Invest Dermatol.

[11] Numata S, Akamatsu H, Akaza N, Yagami A, Nakata S, Matsunaga K (2014). Analysis of facial skin-resident microbiota in Japanese acne patients. Dermatology.

[12] Barnard E, Shi B, Kang D, Craft N, Li H (2016). The balance of metagenomic elements shapes the skin microbiome in acne and health. Sci Rep.

[13] Lomholt HB, Scholz CFP, Brüggemann H, Tettelin H, Kilian M (2017). A comparative study of Cutibacterium (*Propionibacterium*) acnes clones from acne patients and healthy controls. Anaerobe.

[14] Dréno B, Pécastaings S, Corvec S, Veraldi S, Khammari A, Roques C (2018). *Cutibacterium*
*acnes* (*Propionibacterium*
*acnes*) and acne vulgaris: a brief look at the latest updates. J Eur Acad Dermatol Venereol.

[15] Fournière M, Latire T, Souak D, Feuilloley MGJ, Bedoux G (2020). *Staphylococcus*
*epidermidis* and *Cutibacterium*
*acnes*: two major sentinels of skin microbiota and the influence of cosmetics. Microorganisms.

[16] Rozas M, Hart de Ruijter A, Fabrega MJ, Zorgani A, Guell M, Paetzold B (2021). From dysbiosis to healthy skin: major contributions of *Cutibacterium*
*acnes* to skin homeostasis. Microorganisms..

[17] Ferček I, Lugović-Mihić L, Tambić-Andrašević A, Ćesić D, Grginić AG, Bešlić I (2021). Features of the skin microbiota in common inflammatory skin diseases. Life (Basel).

[18] Robert C, Cascella F, Mellai M, Barizzone N, Mignone F, Massa N (2022). Influence of sex on the microbiota of the human face. Microorganisms.

[19] Hu T, Wei Z, Ju Q, Chen W (2021). Sex hormones and acne: state of the art. J Dtsch Dermatol Ges.

[20] Saint-Jean M, Corvec S, Nguyen JM, Le Moigne M, Boisrobert A, Khammari A (2019). Adult acne in women is not associated with a specific type of *Cutibacterium*
*acnes*. J Am Acad Dermatol.

[21] Dagnelie MA, Montassier E, Khammari A, Mounier C, Corvec S, Dréno B (2019). Inflammatory skin is associated with changes in the skin microbiota composition on the back of severe acne patients. Exp Dermatol.

[22] Dagnelie MA, Corvec S, Saint-Jean M, Bourdès V, Nguyen JM, Khammari A (2018). Decrease in diversity of *Propionibacterium*
*acnes* phylotypes in patients with severe acne on the back. Acta Derm Venereol.

[23] Luk NM, Hui M, Lee HC, Fu LH, Liu ZH, Lam LY (2013). Antibiotic-resistant *Propionibacterium*
*acnes* among acne patients in a regional skin centre in Hong Kong. J Eur Acad Dermatol Venereol.

[24] Schneider AM, Nolan ZT, Banerjee K, Paine AR, Cong Z, Gettle SL (2023). Evolution of the facial skin microbiome during puberty in normal and acne skin. J Eur Acad Dermatol Venereol.

[25] Zhou L, Liu X, Li X, He X, Xiong X, Lai J (2022). Epidermal barrier integrity is associated with both skin microbiome diversity and composition in patients with acne vulgaris. Clin Cosmet Investig Dermatol.

[26] Xu X, Ran X, Tang J, Pradhan S, Dai Y, Zhuang K (2021). Skin microbiota in non-inflammatory and inflammatory lesions of acne vulgaris: the underlying changes within the pilosebaceous unit. Mycopathologia.

[27] Lam M, Hu A, Fleming P, Lynde CW (2022). The impact of acne treatment on skin bacterial microbiota: a systematic review. J Cutan Med Surg.

[28] Coughlin CC, Swink SM, Horwinski J, Sfyroera G, Bugayev J, Grice EA (2017). The preadolescent acne microbiome: a prospective, randomized, pilot study investigating characterization and effects of acne therapy. Pediatr Dermatol.

[29] Ahluwalia J, Borok J, Haddock ES, Ahluwalia RS, Schwartz EW, Hosseini D (2019). The microbiome in preadolescent acne assessment and prospective analysis of the influence of benzoyl peroxide. Pediatr Dermatol.

[30] Zhou L, Chen L, Liu X, Huang Y, Xu Y, Xiong X (2022). The influence of benzoyl peroxide on skin microbiota and the epidermal barrier for acne vulgaris. Dermatol Ther.

[31] Chien AL, Tsai J, Leung S, Mongodin EF, Nelson AM, Kang S (2019). Association of systemic antibiotic treatment of acne with skin microbiota characteristics. JAMA Dermatol.

[32] Thompson KG, Rainer BM, Antonescu C, Florea L, Mongodin EF, Kang S (2020). Minocycline and its impact on microbial dysbiosis in the skin and gastrointestinal tract of acne patients. Ann Dermatol.

[33] Park SY, Kim HS, Lee SH, Kim S (2020). Characterization and analysis of the skin microbiota in acne: impact of systemic antibiotics. J Clin Med.

[34] Barbieri JS, Spaccarelli N, Margolis DJ, James WD (2019). Approaches to limit systemic antibiotic use in acne: systemic alternatives, emerging topical therapies, dietary modification, and laser and light-based treatments. J Am Acad Dermatol.

[35] Grech I (2014). Susceptibility profiles of *Propionibacterium*
*acnes* isolated from patients with acne vulgaris. J Glob Antimicrob Resist.

[36] Zhang N, Yuan R, Xin KZ, Lu Z, Ma Y (2019). Antimicrobial susceptibility, biotypes and phylotypes of clinical cutibacterium (Formerly *Propionibacterium*) acnes strains isolated from acne patients an observational study. Dermatol Ther (Heidelb).

[37] Ma Y, Zhang N, Wu S, Huang H, Cao Y (2016). Antimicrobial activity of topical agents against *Propionibacterium*
*acnes*: an in vitro study of clinical isolates from a hospital in Shanghai, China. Front Med.

[38] Zhang N, Lu Z, Ma Y (2017). Draft genome sequences of three multidrug-resistant Cutibacterium (formerly *Propionibacterium*) acnes strains isolated from acne patients, China. J Glob Antimicrob Resist.

[39] Pollock B, Turner D, Stringer MR, Bojar RA, Goulden V, Stables GI (2004). Topical aminolaevulinic acid-photodynamic therapy for the treatment of acne vulgaris: a study of clinical efficacy and mechanism of action. Br J Dermatol.

[40] Ma L, Xiang LH, Yu B, Yin R, Chen L, Wu Y (2013). Low-dose topical 5-aminolevulinic acid photodynamic therapy in the treatment of different severity of acne vulgaris. Photodiagnosis Photodyn Ther.

[41] Serini SM, Cannizzaro MV, Dattola A, Garofalo V, Del Duca E, Ventura A (2019). The efficacy and tolerability of 5-aminolevulinic acid 5% thermosetting gel photodynamic therapy (PDT) in the treatment of mild-to-moderate acne vulgaris. A two-center, prospective assessor-blinded, proof-of-concept study. J Cosmet Dermatol..

[42] Guo Y, Zeng M, Yuan Y, Yuan M, Chen Y, Yu H (2023). Photodynamic therapy treats acne by altering the composition of the skin microbiota. Skin Res Technol.

[43] Yang Y, Tao S, Zeng R, Zheng H, Ge Y (2021). Modulation of skin microbiome in acne patients by aminolevulinic acid-photodynamic therapy. Photodiagnosis Photodyn Ther.

[44] Tao S, Wang Z, Quan C, Ge Y, Qian Q (2021). The effects of ALA-PDT on microbiota in pilosebaceous units of patients with severe acne: a metagenomic study. Photodiagnosis Photodyn Ther.

[45] Simonart T, Dramaix M, De Maertelaer V (2008). Efficacy of tetracyclines in the treatment of acne vulgaris: a review. Br J Dermatol.

[46] Vallerand IA, Lewinson RT, Farris MS, Sibley CD, Ramien ML, Bulloch AGM (2018). Efficacy and adverse events of oral isotretinoin for acne: a systematic review. Br J Dermatol.

[47] Kelhälä HL, Aho VTE, Fyhrquist N, Pereira PAB, Kubin ME, Paulin L (2018). Isotretinoin and lymecycline treatments modify the skin microbiota in acne. Exp Dermatol.

[48] Zhang L, Shao X, Chen Y, Wang J, Ariyawati A, Zhang Y (2022). 30% supramolecular salicylic acid peels effectively treats acne vulgaris and reduces facial sebum. J Cosmet Dermatol.

[49] Shao X, Chen Y, Zhang L, Zhang Y, Ariyawati A, Chen T (2023). Effect of 30% supramolecular salicylic acid peel on skin microbiota and inflammation in patients with moderate-to-severe acne vulgaris. Dermatol Ther (Heidelb).

[50] Bilal H, Xiao Y, Khan MN, Chen J, Wang Q, Zeng Y (2023). Stabilization of acne vulgaris-associated microbial dysbiosis with 2% supramolecular salicylic acid. Pharmaceuticals (Basel).

[51] Pécastaings S, Roques C, Nocera T, Peraud C, Mengeaud V, Khammari A (2023). Myrtus communis and celastrol enriched plant cell culture extracts control together the pivotal role of *Cutibacterium*
*acnes* and inflammatory pathways in acne. J Eur Acad Dermatol Venereol.

[52] Pécastaings S, Roques C, Nocera T, Peraud C, Mengeaud V, Khammari A (2018). Characterisation of *Cutibacterium*
*acnes* phylotypes in acne and in vivo exploratory evaluation of Myrtacine. J Eur Acad Dermatol Venereol.

[53] Isard O, Knol AC, Ariès MF, Nguyen JM, Khammari A, Castex-Rizzi N (2011). Propionibacterium acnes activates the IGF-1/IGF-1R system in the epidermis and induces keratinocyte proliferation. J Invest Dermatol.

[54] Akaza N, Akamatsu H, Kishi M, Mizutani H, Ishii I, Nakata S (2009). Effects of *Propionibacterium*
*acnes* on various mRNA expression levels in normal human epidermal keratinocytes in vitro. J Dermatol.

[55] Bolla BS, Erdei L, Urbán E, Burián K, Kemény L, Szabó K (2020). Cutibacterium acnes regulates the epidermal barrier properties of HPV-KER human immortalized keratinocyte cultures. Sci Rep.

[56] Megyeri K, Orosz L, Bolla S, Erdei L, Rázga Z, Seprényi G (2018). Propionibacterium acnes induces autophagy in keratinocytes: involvement of multiple mechanisms. J Invest Dermatol.

[57] Grange PA, Chéreau C, Raingeaud J, Nicco C, Weill B, Dupin N (2009). Production of superoxide anions by keratinocytes initiates *P*. *acnes*-induced inflammation of the skin. PLoS Pathog..

[58] Schaller M, Loewenstein M, Borelli C, Jacob K, Vogeser M, Burgdorf WH (2005). Induction of a chemoattractive proinflammatory cytokine response after stimulation of keratinocytes with *Propionibacterium*
*acnes* and coproporphyrin III. Br J Dermatol.

[59] Ramage G, Tunney MM, Patrick S, Gorman SP, Nixon JR (2003). Formation of Propionibacterium acnes biofilms on orthopaedic biomaterials and their susceptibility to antimicrobials. Biomaterials.

[60] Bayston R, Ashraf W, Barker-Davies R (2007). Biofilm formation by *Propionibacterium*
*acnes* on biomaterials in vitro and in vivo: impact on diagnosis and treatment. J Biomed Mater Res A.

[61] Holmberg A, Lood R, Mörgelin M, Söderquist B, Holst E, Collin M (2009). Biofilm formation by *Propionibacterium*
*acnes* is a characteristic of invasive isolates. Clin Microbiol Infect.

[62] Loss M, Thompson KG, Agostinho-Hunt A, James GA, Mongodin EF, Rosenthal I (2021). Noninflammatory comedones have greater diversity in microbiome and are more prone to biofilm formation than inflammatory lesions of acne vulgaris. Int J Dermatol.

[63] Kuehnast T, Cakar F, Weinhäupl T, Pilz A, Selak S, Schmidt MA (2018). Comparative analyses of biofilm formation among different *Cutibacterium*
*acnes* isolates. Int J Med Microbiol.

[64] Cavallo I, Sivori F, Truglio M, De Maio F, Lucantoni F, Cardinali G (2022). Skin dysbiosis and *Cutibacterium*
*acnes* biofilm in inflammatory acne lesions of adolescents. Sci Rep.

[65] Zeng R, Xu H, Liu Y, Du L, Duan Z, Tong J (2019). miR-146a inhibits biofilm-derived *Cutibacterium*
*acnes*-induced inflammatory reactions in human keratinocytes. J Invest Dermatol.

[66] Sugisaki H, Yamanaka K, Kakeda M, Kitagawa H, Tanaka K, Watanabe K (2009). Increased interferon-gamma, interleukin-12p40 and IL-8 production in *Propionibacterium*
*acnes*-treated peripheral blood mononuclear cells from patient with acne vulgaris: host response but not bacterial species is the determinant factor of the disease. J Dermatol Sci.

[67] Lomholt HB, Kilian M (2010). Population genetic analysis of *Propionibacterium*
*acnes* identifies a subpopulation and epidemic clones associated with acne. PLoS ONE.

[68] Kolar SL, Tsai CM, Torres J, Fan X, Li H, Liu GY (2019). *Propionibacterium*
*acnes*-induced immunopathology correlates with health and disease association. JCI Insight.

[69] Yu Y, Champer J, Agak GW, Kao S, Modlin RL, Kim J (2016). Different *Propionibacterium*
*acnes* phylotypes induce distinct immune responses and express unique surface and secreted proteomes. J Invest Dermatol.

[70] Agak GW, Qin M, Nobe J, Kim MH, Krutzik SR, Tristan GR (2014). *Propionibacterium*
*acnes* induces an IL-17 response in acne vulgaris that is regulated by Vitamin A and Vitamin D. J Invest Dermatol.

[71] Agak GW, Kao S, Ouyang K, Qin M, Moon D, Butt A (2018). Phenotype and antimicrobial activity of Th17 cells induced by propionibacterium acnes strains associated with healthy and acne skin. J Invest Dermatol.

[72] Kistowska M, Meier B, Proust T, Feldmeyer L, Cozzio A, Kuendig T (2015). *Propionibacterium*
*acnes* promotes Th17 and Th17/Th1 responses in acne patients. J Invest Dermatol.

[73] Kistowska M, Gehrke S, Jankovic D, Kerl K, Fettelschoss A, Feldmeyer L (2014). IL-1β drives inflammatory responses to propionibacterium acnes in vitro and in vivo. J Invest Dermatol.

[74] Qin M, Pirouz A, Kim MH, Krutzik SR, Garbán HJ, Kim J (2014). *Propionibacterium*
*acnes* Induces IL-1β secretion via the NLRP3 inflammasome in human monocytes. J Invest Dermatol.

[75] Li ZJ, Choi DK, Sohn KC, Seo MS, Lee HE, Lee Y (2014). *Propionibacterium*
*acnes* activates the NLRP3 inflammasome in human sebocytes. J Invest Dermatol.

[76] Kim J, Ochoa MT, Krutzik SR, Takeuchi O, Uematsu S, Legaspi AJ (2002). Activation of toll-like receptor 2 in acne triggers inflammatory cytokine responses. J Immunol.

[77] Shibata M, Katsuyama M, Onodera T, Ehama R, Hosoi J, Tagami H (2009). Glucocorticoids enhance Toll-like receptor 2 expression in human keratinocytes stimulated with *Propionibacterium*
*acnes* or proinflammatory cytokines. J Invest Dermatol.

[78] Jugeau S, Tenaud I, Knol AC, Jarrousse V, Quereux G, Khammari A (2005). Induction of toll-like receptors by *Propionibacterium*
*acnes*. Br J Dermatol.

[79] Nagy I, Pivarcsi A, Koreck A, Széll M, Urbán E, Kemény L (2005). Distinct strains of *Propionibacterium*
*acnes* induce selective human beta-defensin-2 and interleukin-8 expression in human keratinocytes through toll-like receptors. J Invest Dermatol.

[80] Huang YC, Yang CH, Li TT, Zouboulis CC, Hsu HC (2015). Cell-free extracts of *Propionibacterium*
*acnes* stimulate cytokine production through activation of p38 MAPK and Toll-like receptor in SZ95 sebocytes. Life Sci.

[81] Wang Y, Hata TR, Tong YL, Kao MS, Zouboulis CC, Gallo RL (2018). The anti-inflammatory activities of *Propionibacterium*
*acnes* CAMP factor-targeted acne vaccines. J Invest Dermatol.

[82] Lheure C, Grange PA, Ollagnier G, Morand P, Désiré N, Sayon S (2016). TLR-2 recognizes *Propionibacterium*
*acnes* CAMP factor 1 from highly inflammatory strains. PLoS ONE.

[83] Hu T, Pan Z, Yu Q, Mo X, Song N, Yan M (2016). Benzo(a)pyrene induces interleukin (IL)-6 production and reduces lipid synthesis in human SZ95 sebocytes via the aryl hydrocarbon receptor signaling pathway. Environ Toxicol Pharmacol.

[84] Napolitano M, Fabbrocini G, Martora F, Picone V, Morelli P, Patruno C (2021). Role of aryl hydrocarbon receptor activation in inflammatory chronic skin diseases. Cells.

[85] Hu T, Wang D, Yu Q, Li L, Mo X, Pan Z (2016). Aryl hydrocarbon receptor negatively regulates lipid synthesis and involves in cell differentiation of SZ95 sebocytes in vitro. Chem Biol Interact.

[86] Muku GE, Blazanin N, Dong F, Smith PB, Thiboutot D, Gowda K (2019). Selective Ah receptor ligands mediate enhanced SREBP1 proteolysis to restrict lipogenesis in sebocytes. Toxicol Sci.

[87] Hou XX, Chen G, Hossini AM, Hu T, Wang L, Pan Z (2019). Aryl hydrocarbon receptor modulates the expression of TNF-α and IL-8 in human sebocytes via the MyD88-p65NF-κB/p38MAPK signaling pathways. J Innate Immun.

[88] Cao K, Chen G, Chen W, Hou X, Hu T, Lu L (2021). Formalin-killed *Propionibacterium*
*acnes* activates the aryl hydrocarbon receptor and modifies differentiation of SZ95 sebocytes in vitro. Eur J Dermatol.

[89] Li F, Lin L, He Y, Sun G, Dong D, Wu B (2022). BMAL1 regulates *Propionibacterium*
*acnes*-induced skin inflammation via REV-ERBα in mice. Int J Biol Sci.

[90] Erdei L, Bolla BS, Bozó R, Tax G, Urbán E, Kemény L (2018). TNIP1 regulates *Cutibacterium*
*acnes*-induced innate immune functions in epidermal keratinocytes. Front Immunol.

[91] Erdei L, Bolla BS, Bozó R, Tax G, Urbán E, Burián K (2021). Tumour necrosis factor alpha-induced protein 3 negatively regulates *Cutibacterium*
*acnes*-induced innate immune events in epidermal keratinocytes. Acta Derm Venereol..

[92] Yu Y, Shen Y, Zhang S, Wang N, Luo L, Zhu X (2022). Suppression of *Cutibacterium*
*acnes*-mediated inflammatory reactions by fibroblast growth factor 21 in skin. Int J Mol Sci.

[93] Nishijima S, Kurokawa I, Katoh N, Watanabe K (2000). The bacteriology of acne vulgaris and antimicrobial susceptibility of Propionibacterium acnes and Staphylococcus epidermidis isolated from acne lesions. J Dermatol.

[94] Ahle CM, Stødkilde K, Poehlein A, Bömeke M, Streit WR, Wenck H (2022). Interference and co-existence of *staphylococci* and *Cutibacterium*
*acnes* within the healthy human skin microbiome. Commun Biol.

[95] Wang Y, Kuo S, Shu M, Yu J, Huang S, Dai A (2014). Staphylococcus epidermidis in the human skin microbiome mediates fermentation to inhibit the growth of *Propionibacterium*
*acnes*: implications of probiotics in acne vulgaris. Appl Microbiol Biotechnol.

[96] Xia X, Li Z, Liu K, Wu Y, Jiang D, Lai Y (2016). Staphylococcal LTA-Induced miR-143 Inhibits *Propionibacterium*
*acnes*-mediated inflammatory response in skin. J Invest Dermatol.

[97] Choi EJ, Lee HG, Bae IH, Kim W, Park J, Lee TR (2018). *Propionibacterium*
*acnes*-derived extracellular vesicles promote acne-like phenotypes in human epidermis. J Invest Dermatol.

[98] Lee SE, Kim JM, Jeong SK, Jeon JE, Yoon HJ, Jeong MK (2010). Protease-activated receptor-2 mediates the expression of inflammatory cytokines, antimicrobial peptides, and matrix metalloproteinases in keratinocytes in response to Propionibacterium acnes. Arch Dermatol Res.

[99] Smith TM, Gilliland K, Clawson GA, Thiboutot D (2008). IGF-1 induces SREBP-1 expression and lipogenesis in SEB-1 sebocytes via activation of the phosphoinositide 3-kinase/Akt pathway. J Invest Dermatol.

[100] Gu H, An HJ, Gwon MG, Bae S, Zouboulis CC, Park KK (2022). The effects of synthetic SREBP-1 and PPAR-γ decoy oligodeoxynucleotide on acne-like disease in vivo and in vitro via lipogenic regulation. Biomolecules.

[101] Lee SE, Kim JM, Jeong SK, Choi EH, Zouboulis CC, Lee SH (2015). Expression of protease-activated receptor-2 in SZ95 sebocytes and its role in sebaceous lipogenesis, inflammation, and innate immunity. J Invest Dermatol.

[102] Borelli C, Merk K, Schaller M, Jacob K, Vogeser M, Weindl G (2006). In vivo porphyrin production by *P*. *acnes* in untreated acne patients and its modulation by acne treatment. Acta Derm Venereol..

[103] Johnson T, Kang D, Barnard E, Li H (2016). Strain-level differences in porphyrin production and regulation in *Propionibacterium*
*acnes* elucidate disease Associations. mSphere..

[104] Barnard E, Johnson T, Ngo T, Arora U, Leuterio G, McDowell A (2020). Porphyrin production and regulation in cutaneous *Propionibacteria*. mSphere..

[105] Kang D, Shi B, Erfe MC, Craft N, Li H (2015). Vitamin B12 modulates the transcriptome of the skin microbiota in acne pathogenesis. Sci Transl Med..

[106] Spittaels KJ, van Uytfanghe K, Zouboulis CC, Stove C, Crabbé A, Coenye T (2021). Porphyrins produced by acneic *Cutibacterium*
*acnes* strains activate the inflammasome by inducing K+ leakage. iScience..

[107] Tax G, Urbán E, Palotás Z, Puskás R, Kónya Z, Bíró T (2016). Propionic acid produced by *Propionibacterium*
*acnes* strains contributes to their pathogenicity. Acta Derm Venereol.

[108] Lim HJ, Park IS, Jie EY, Ahn WS, Kim SJ, Jeong SI (2020). Anti-inflammatory activities of an extract of in vitro grown adventitious shoots of *Toona*
*sinensis* in LPS-treated RAW264.7 and *Propionibacterium*
*acnes*-treated HaCaT cells. Plants (Basel)..

[109] Grange PA, Raingeaud J, Calvez V, Dupin N (2009). Nicotinamide inhibits *Propionibacterium*
*acnes*-induced IL-8 production in keratinocytes through the NF-kappaB and MAPK pathways. J Dermatol Sci.

[110] Zhu T, Fang F, Sun D, Yang S, Zhang X, Yu X (2020). Piceatannol inhibits *P*. *acnes*-induced keratinocyte proliferation and migration by downregulating oxidative stress and the inflammatory response. Inflammation..

[111] Oh Y, Hwang HJ, Yang H, Kim JH, Park JHY, Kim JE (2020). Orobol, a derivative of genistein, inhibits heat-killed *Propionibacterium*
*acnes*-induced inflammation in HaCaT keratinocytes. J Microbiol Biotechnol.

[112] Yang G, Lee HE, Yeon SH, Kang HC, Cho YY, Lee HS (2018). Licochalcone A attenuates acne symptoms mediated by suppression of NLRP3 inflammasome. Phytother Res.

[113] Guo M, An F, Yu H, Wei X, Hong M, Lu Y (2017). Comparative effects of schisandrin A, B, and C on *Propionibacterium*
*acnes*-induced, NLRP3 inflammasome activation-mediated IL-1β secretion and pyroptosis. Biomed Pharmacother.

[114] Fang F, Xie Z, Quan J, Wei X, Wang L, Yang L (2020). Baicalin suppresses Propionibacterium acnes-induced skin inflammation by downregulating the NF-κB/MAPK signaling pathway and inhibiting activation of NLRP3 inflammasome. Braz J Med Biol Res.

[115] Yang S, Jiang Y, Yu X, Zhu L, Wang L, Mao J (2021). Polyphyllin I inhibits *Propionibacterium*
*acnes*-induced IL-8 secretion in HaCaT cells by downregulating the CD36/NOX1/ROS/NLRP3/IL-1β pathway. Evid Based Complement Alternat Med.

[116] Zhu T, Wu W, Yang S, Li D, Sun D, He L (2019). Polyphyllin I inhibits *Propionibacterium*
*acnes*-induced inflammation in vitro. Inflammation.

[117] Fernández JR, Webb C, Rouzard K, Healy J, Tamura M, Voronkov M (2018). SIG1459: A novel phytyl-cysteine derived TLR2 modulator with in vitro and clinical anti-acne activity. Exp Dermatol.

[118] Fernandéz JR, Rouzard K, Voronkov M, Feng X, Stock JB, Stock M (2012). SIG1273: a new cosmetic functional ingredient to reduce blemishes and *Propionibacterium*
*acnes* in acne prone skin. J Cosmet Dermatol.

[119] Chen KC, Yang CH, Li TT, Zouboulis CC, Huang YC (2019). Suppression of Propionibacterium acnes-stimulated proinflammatory cytokines by Chinese bayberry extracts and its active constituent myricetin in human sebocytes in vitro. Phytother Res.

[120] Lim HJ, Kang SH, Song YJ, Jeon YD, Jin JS (2021). Inhibitory effect of quercetin on *Propionibacterium*
*acnes*-induced skin inflammation. Int Immunopharmacol.

[121] De Canha MN, Komarnytsky S, Langhansova L, Lall N (2020). Exploring the anti-acne potential of Impepho [*Helichrysum*
*odoratissimum* (L.) Sweet] to Combat *Cutibacterium*
*acnes* virulence. Front Pharmacol..

[122] Dell'Annunziata F, Cometa S, Della Marca R, Busto F, Folliero V, Franci G (2022). In vitro antibacterial and anti-inflammatory activity of arctostaphylos uva-ursi leaf extract against *Cutibacterium*
*acnes*. Pharmaceutics.

[123] Kim YG, Lee JH, Park S, Lee J (2022). The anticancer agent 3,3'-diindolylmethane inhibits multispecies biofilm formation by acne-causing bacteria and *Candida*
*albicans*. Microbiol Spectr.

[124] Attia-Vigneau J, Barreau M, Le Toquin E, Feuilloley MGJ, Loing E, Lesouhaitier O (2022). Polylysine dendrigraft is able to differentially impact *Cutibacterium*
*acnes* strains preventing acneic skin. Exp Dermatol.

[125] Jin S, Lee MY (2018). *Kaempferia*
*parviflora* extract as a potential anti-acne agent with anti-inflammatory, sebostatic and anti- propionibacterium acnes activity. Int J Mol Sci.

[126] Tollenaere M, Boira C, Chapuis E, Lapierre L, Jarrin C, Robe P (2022). Action of *Mangifera*
*indica* leaf extract on acne-prone skin through sebum harmonization and targeting *C*. *acnes*. Molecules..

[127] Gu H, An HJ, Gwon MG, Bae S, Leem J, Lee SJ (2022). Bee venom and its major component melittin attenuated *Cutibacterium*
*acnes*- and IGF-1-Induced acne vulgaris via inactivation of Akt/mTOR/SREBP signaling pathway. Int J Mol Sci.

[128] Ryan-Kewley AE, Williams DR, Hepburn N, Dixon RA (2017). Non-antibiotic isotretinoin treatment differentially controls *Propionibacterium*
*acnes* on skin of acne patients. Front Microbiol.

[129] Batra R, Sadhasivam S, Saini S, Gupta S, Bisen RKS, Sinha M (2020). Efficacy and safety of VB-1953 topical gel in non-responder acne patients with clindamycin-resistant cutibacterium acnes. Drugs R D.

[130] Nakatsuji T, Liu YT, Huang CP, Zoubouis CC, Gallo RL, Huang CM (2008). Antibodies elicited by inactivated propionibacterium acnes-based vaccines exert protective immunity and attenuate the IL-8 production in human sebocytes: relevance to therapy for acne vulgaris. J Invest Dermatol.

[131] Lee YJ, Choi HJ, Kang TW, Kim HO, Chung MJ, Park YM (2008). CBT-SL5, a bacteriocin from *Enterococcus*
*faecalis*, suppresses the expression of interleukin-8 induced by *Propionibacterium*
*acnes* in cultured human keratinocytes. J Microbiol Biotechnol.

[132] Kang BS, Seo JG, Lee GS, Kim JH, Kim SY, Han YW (2009). Antimicrobial activity of enterocins from *Enterococcus*
*faecalis* SL-5 against *Propionibacterium*
*acnes*, the causative agent in acne vulgaris, and its therapeutic effect. J Microbiol.

[133] Han HS, Shin SH, Choi BY, Koo N, Lim S, Son D (2022). A split face study on the effect of an anti-acne product containing fermentation products of *Enterococcus*
*faecalis* CBT SL-5 on skin microbiome modification and acne improvement. J Microbiol.

[134] Mottin VHM, Suyenaga ES (2018). An approach on the potential use of probiotics in the treatment of skin conditions: acne and atopic dermatitis. Int J Dermatol.

[135] Goodarzi A, Mozafarpoor S, Bodaghabadi M, Mohamadi M (2020). The potential of probiotics for treating acne vulgaris: a review of literature on acne and microbiota. Dermatol Ther.

[136] Woo TE, Sibley CD (2020). The emerging utility of the cutaneous microbiome in the treatment of acne and atopic dermatitis. J Am Acad Dermatol.

[137] O'Neill AM, Nakatsuji T, Hayachi A, Williams MR, Mills RH, Gonzalez DJ (2020). Identification of a human skin commensal bacterium that selectively kills *Cutibacterium*
*acnes*. J Invest Dermatol.

[138] Karoglan A, Paetzold B, Pereira de Lima J, Brüggemann H, Tüting T, Schanze D (2019). Safety and efficacy of topically applied selected *Cutibacterium*
*acnes* strains over five weeks in patients with acne vulgaris an open-label, pilot study. Acta Derm Venereol..

[139] Lebeer S, Oerlemans EFM, Claes I, Henkens T, Delanghe L, Wuyts S (2022). Selective targeting of skin pathobionts and inflammation with topically applied lactobacilli. Cell Rep Med.

[140] Marito S, Keshari S, Huang CM (2020). PEG-8 Laurate fermentation of *Staphylococcus*
*epidermidis* reduces the required dose of clindamycin against *Cutibacterium*
*acnes*. Int J Mol Sci.

[141] Marito S, Keshari S, Traisaeng S, My DTT, Balasubramaniam A, Adi P (2021). Electricity-producing *Staphylococcus*
*epidermidis* counteracts *Cutibacterium*
*acnes*. Sci Rep.

[142] Yang AJ, Marito S, Yang JJ, Keshari S, Chew CH, Chen CC (2018). A microtube array membrane (MTAM) encapsulated live fermenting *Staphylococcus*
*epidermidis* as a skin probiotic patch against *Cutibacterium*
*acnes*. Int J Mol Sci.

[143] Garcia-Cancino A, Albarracin L, Espinoza-Monje M, Campos-Martin J, Garcia-Castillo V, Nakano Y (2019). Draft genome sequence of *Weissella*
*viridescens* UCO-SMC3, Isolated from the Slime of Helix aspersa Müller Snails. Microbiol Resour Announc.

[144] Espinoza-Monje M, Campos J, Alvarez Villamil E, Jerez A, Dentice Maidana S, Elean M (2021). Characterization of *Weissella*
*viridescens* UCO-SMC3 as a potential probiotic for the skin its beneficial role in the pathogenesis of acne vulgaris. Microorganisms.

[145] Sathikulpakdee S, Kanokrungsee S, Vitheejongjaroen P, Kamanamool N, Udompataikul M, Taweechotipatr M (2022). Efficacy of probiotic-derived lotion from *Lactobacillus*
*paracasei* MSMC 39–1 in mild to moderate acne vulgaris, randomized controlled trial. J Cosmet Dermatol.

[146] Cui H, Guo C, Wang Q, Feng C, Duan Z (2022). A pilot study on the efficacy of topical lotion containing anti-acne postbiotic in subjects with mild -to -moderate acne. Front Med (Lausanne).

